# Guiding atrial fibrillation ablation combined with left atrial appendage occlusion procedure by fluoroscopy with or without transesophageal echocardiography achieved comparable outcomes

**DOI:** 10.1002/clc.23993

**Published:** 2023-03-09

**Authors:** Weilun Meng, Xiang Li, Zhongyuan Ren, Yixing Zheng, Jingying Zhang, Haotian Yang, Rong Guo, Hailing Li, Jun Zhang, Yiqian Wang, Peng Jia, Dongdong Zhao, Yawei Xu

**Affiliations:** ^1^ Department of Cardiology, Shanghai Tenth People's Hospital Tongji University School of Medicine Shanghai China

**Keywords:** catheter ablation, combined procedure, digital subtraction angiography, left atrial appendage occlusion, transesophageal echocardiography

## Abstract

**Background:**

Atrial fibrillation (AF) is the most common arrhythmia and can be treated with catheter ablation (CA) combined with left atrial appendage occlusion (LAAO). The study is designed to compare the safety and efficacy of guiding the combined procedure by digital subtraction angiography (DSA) with or without transesophageal echocardiography (TEE).

**Methods:**

From February 2019 to December 2020, 138 patients with nonvalvular AF who underwent CA combined with LAAO procedure were consecutively included, and two cohorts were built according to intraprocedural guidance (DSA or DSA with TEE). Periprocedural and follow‐up outcomes were compared with investigate the feasibility and safety between the two cohorts.

**Results:**

71 patients and 67 patients were included in the DSA cohort and TEE cohort, respectively. Age and gender were comparable, despite the TEE cohort having a higher proportion of persistent AF (37 [55.2%] vs. 26 [36.6%]) and hemorrhage history (9 [13.4%] vs. 0). The procedure time of the DSA cohort was significantly reduced (95.7 ± 27.6 vs. 108.9 ± 30.3 min, *p* = .018), with a nonsignificant longer fluoroscopic time (15.2 ± 5.4 vs. 14.4 ± 7.1 min, *p* = .074). And the overall incidence of peri‐procedural complications was similar between cohorts. After an average of 24 months of clinical follow‐up, only three patients in the TEE cohort had ≤3 mm residual flow (*p* = .62). Kaplan–Meier estimates showed nonsignificant differences between the cohorts for freedom from atrial arrhythmia (log‐rank *p* = .964) and major adverse cardiovascular events (log‐rank *p* = .502).

**Conclusions:**

Compared with DSA and TEE guidance, DSA‐guided combined procedure could shorten the procedural time, while achieving similar periprocedural and long‐term feasibility and safety.

## Graphical abstract

One hundred and thirty eight patients with nonvalvular atrial fibrillation who underwent the combined procedure were consecutively included, and two cohorts were built according to intraprocedural guidance. We found that compared with digital subtraction angiography (DSA) and transesophageal echocardiography guidance, DSA‐guided combined procedure could shorten the procedural time while achieving similar periprocedural and long‐term feasibility and safety.

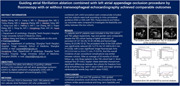

## 1 INTRODUCTION

Atrial fibrillation (AF) is the most common chronic degenerative arrhythmia with a 0.65% prevalence over 10 million in China.^1^ AF increases the risk of heart failure and stroke, which seriously affects the life quality of patients.^2^, ^3^, ^4^ However, due to low compliance of adequate pharmacological anticoagulation and rhythm control in China, AF has become a serious issue in the Chinese population.^5^ In terms of limitation of pharmacological stroke prevention, 2019 AHA/ACC guidelines (IIB) recommended left atrial appendage occlusion (LAAO) for AF patients having contraindication for long‐term oral anticoagulant (OAC) treatment.^6^ Catheter ablation (CA) with minimal lesions has been recommended to treat drug‐refractory AF since pulmonary vein isolation (PVI) was reported to effectively control AF.^7^, ^8^, ^9^ To control AF and prevent stroke, Swans et al.^10^ reported the first 30 symptomatic drug‐refractory AF patients who were at a high risk of stroke and had contraindications for OACs with successful CA combined with LAAO in a single procedure. Many other following studies have proved CA combined with LAAO safe and effective.^11^, ^12^, ^13^, ^14^

In the LAAO procedure, it is crucial to ensure the LAAO device to be delivered and deployed in the desired position under safe and feasible evaluation of intraprocedural device surveillance, which can furthest diminish peri‐device leakage (PDL) and avoid procedure‐related complications.^15^ Transesophageal echocardiography (TEE) has been recommended by EHRA/EAPCI expert consensus as the standard method to guide LAAO procedures under general anesthesia.^6^ However, TEE application is limited by a dedicated anesthetic and echocardiographic team, increasing the risk of general anesthesia and esophageal injury.^16^ By contrast, with the increasing maturity of LAAO technology, there have been many articles describing the safety and feasibility of the LAAO using digital subtraction angiography (DSA) alone.^16^, ^17^, ^18^, ^19^ Nevertheless, the current articles about CA combined with LAAO procedure with DSA alone have not been reported. In the present study, we compared the feasibility and safety of intraprocedural guidance of combined procedure with DSA alone to TEE and DSA.

## 2 METHOD

### 2.1 Study population

We retrospectively collected nonvalvular AF patients who were hospitalized in Shanghai Tenth People's Hospital and underwent CA combined with LAAO procedure from February 2019 to December 2020, and we divided them into two consecutive cohorts according to intra‐procedural DSA with TEE guidance or DSA guidance alone. All of the patients were included in the registered retrospective study, “Combining Left Atrial Appendage Closure with Cryoballoon Ablation in Chinese Population” (hereafter referred to as CLACBAC; registry No. NCT04185142). In the CLACBAC study, we consecutively collected all patients who underwent the combined procedure and established the database. And in this study, we enrolled the subjects according to the inclusion and exclusion criteria. According to the 2019 AHA/ACC/HRS Guideline for the Management of Patients with AF,^6^ AF is defined in this study as 12‐lead electrocardiography (ECG) or 24‐h dynamic ECG (Holter) recorded with absolutely varying RR intervals and obvious P wave absence lasting at least 30 s. Paroxysmal atrial fibrillation is defined as AF that can be converted to sinus rhythm spontaneously or by the intervention (electro version or antiarrhythmic drugs) within 7 days. Persistent AF (persAF) is defined as AF lasting more than 7 days, including AF that has been terminated after 7 days. The inclusion criteria for the combined procedure patients include at least one of the following three items: (1) CHA2DS2‐VASc Score ≥ 2 and/or HAS‐BLED Score ≥ 3; (2) contraindications for long‐term OACs, including major active bleeding disorders, hereditary bleeding disorders, or serious side effects of OACs; (3) refusal of OACs according to personal willingness despite comprehensive explanation. Exclusion criteria include: (1) the presence of thrombus in the left atrium (LA) or LAA confirmed by TEE examination; (2) LA diameter greater than 65 mm measured by TTE or LAA opening diameter greater than 35 mm measured by TEE; three preprocedural pericardial effusion of medium and above (>4 mm); (4) hemorrhagic or ischemic stroke within 30 days; (5) hemodynamic instability (blood pressure lower than 90/60 mmHg); (6) active hemorrhagic disease(s). Every patient fully understanded the risks of the procedure and related complications and signed an informed consent form before the procedure. Our study complies with the declaration of Helsinki and was approved by the ethics committee of Shanghai Tenth People′s Hospital.

### 2.2 Preprocedural preparation

Before the procedure, we completed the examination of heart rate, blood pressure, breathing, body temperature, 12‐lead ECG and 24‐h Holter, blood routine, liver and kidney function, electrolytes, coagulation function, myocardial markers, hepatitis B tuberculosis, and other infectious diseases and improved in time abnormal indicators. Furthermore, chest X‐ray and echocardiography were inspected to check obvious structural abnormalities and cranial CT was examinated to assess the risk of stroke. TEE (GE Vivid E9 and Siemens ACUSON SC2000) was performed with an empty belly 24 h before the procedure to check for LA and LAA thrombosis. The LAA morphology was observed at 0°/45°/90°/135° and the LAA diameter, depth, the size of the inner and outer orifice, and evaluate whether there was pericardial effusion and abnormal heart structure.

Patients taking new OACs novel oral anticoagulants (NOACs) stopped NOACs 24 h before the procedure; while for patients taking warfarin orally, the preprocedural international normalized ratio needed to be adjusted to 2.0–3.0. For patients who took antiarrhythmic drugs before the procedure, the AADs needed to be stopped before five drug metabolism half‐lives.

### 2.3 Combined procedure

Patients need fasting for at least 6 h before the procedure. The CA combined with LAAO procedure was performed in the cardiac interventional catheterization room under the monitoring of blood pressure, heart rate, and breathing throughout the whole process. During the combined procedure, the LAAO device was implanted instantly after CA. “Ablation time” was calculated from the time of venipuncture to the time of accomplishment of PVI, and “LAAO duration” was started from the time of advancing the sheath of LAAO to the time of venipuncture access was sutured. Details of the combined procedure have been reported elsewhere.^20^

For DSA and TEE guidance cohort, the combined procedure was performed under local anesthesia with medicine sedation with the standard protocol.^15^ Once the LAAO device was released, TEE at 0°/45°/90°/135° views and DSA at right anterior oblique (RAO) 30°, caudal (CAU) 20°/cranial (CRAN) 20° were obtained to evaluate position of the device and whether there was pericardial effusion.

**Figure 1:**
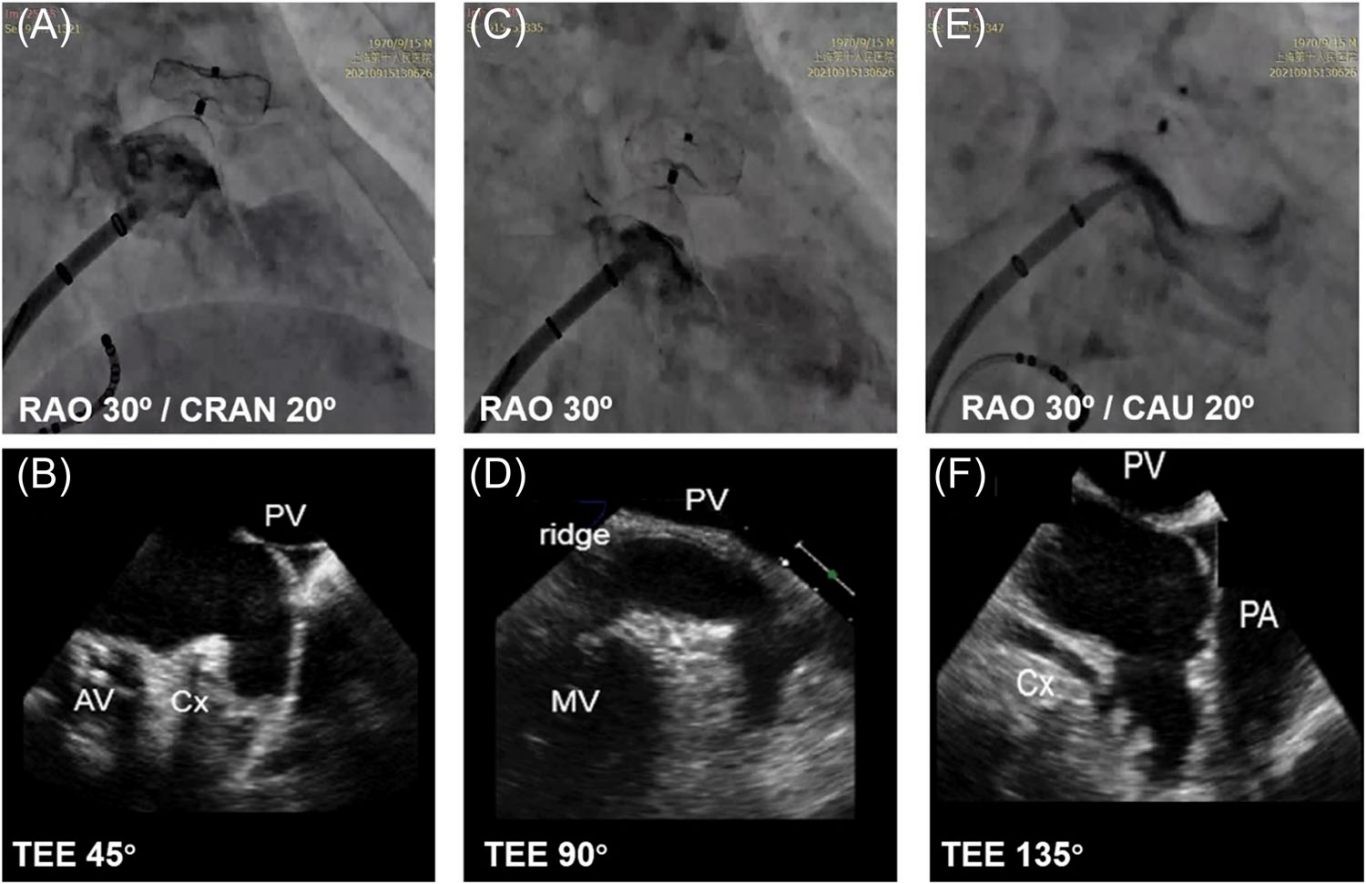
The routine views of digital subtraction angiography and TEE for evaluation of the combined procedure. (A, B): LAA angiography at RAO 30°/CARN 20°corrected with TEE 45°; (C, D): LAA angiography at RAO 30° corrected with TEE 90°; (E, F): LAA angiography at RAO 30°/CAU 20° corrected with TEE 135°. CAU, caudal; CRAN, cranial; RAO, right anterior oblique; TEE, transesophageal echocardiography.

For the DSA guidance alone cohort, LAAO was performed under local anesthesia with DSA. The LAAO delivery sheath and pigtail catheter could be inserted through the femoral vein access established by CA. Before and after the release of the occluder, we mainly used three DSA observation positions for guidance, including RAO 30°/CRAN 20°, RAO 30°, and RAO 30°/CAU 20° which were corrected with the TEE 45°, 90°, 135° view, respectively (Figure 1). Before the release of the occluder, RAO 30°/CAU 20° and RAO 30°/CRAN 20° were located to observe the long and short axis of the LAA as the reference for selecting the occluder model. After the release of the occluder, RAO 30°/CAU 20° was located to evaluate the LAA anterior superior, posterior inferior shunt, and RAO 30°/CRAN 20° was located to evaluate the LAA posterior superior, anterior inferior shunt and whether there was edema in the pulmonary vein spine at the upper edge of the LAA, which might affect the orifice of the pulmonary vein. RAO 30° view was mainly used to evaluate whether there was a shunt in the superior and inferior LAA and the effect on the mitral valve annulus.

### 2.4 Postprocedural management and follow‐up

After the procedure, bilateral femoral vein puncture needed compression bandaging and patients kept bed immobilization for 6–8 h. What's more, NOACs (rivaroxaban or dabigatran) were given and oral antiarrhythmic drugs were given to maintain sinus rhythm or control ventricular rate according to the heart rate. Proton pump inhibitors were given routinely to protect the gastrointestinal mucosa. According to the vital signs, the patients were hospitalized for observation 1–2 days after the procedure and then discharged for follow‐up. After the procedure, an anticoagulation program is formulated based on the follow‐up results. The standard anticoagulant treatment program was as follows: (i) OACs (warfarin or NOACs) will be taken in the first 3 months; (ii) if no LAAO‐related events occur during the first 3 months follow‐up, OAC was changed to dual antiplatelet drug therapy, and lifelong monoclonal antiplatelet drug therapy at the 6 month; (iii) if LAAO‐related events occur, oral anticoagulants are needed to take for life.

All patients required outpatient follow‐up at 1, 3, 6, and 12 months, and every year after the procedure. In addition, a telephone follow‐up was conducted every 3 months to observe the clinical results. The patient's vital signs and medical history were routinely recorded in the outpatient follow‐up, and ECG monitoring was carried out for 24 h or 7 days. At the 3rd month follow‐up, the patient was required to undergo a TEE/CTA examination to assess the occlusion of the LAA. The assessment included the location of the occluder, residual flow, and device‐related thrombotic events. If there are LAAO‐related events (occluder displacement, residual flow >3 mm, and device‐related thrombosis), another TEE inspection is required in the 12th month.

The primary endpoint was the recurrence of atrial arrhythmia (AA) and stroke. AA recurrence was defined as AF, atrial flutter, or atrial tachycardia lasting more than 30 s after 3 months. The first 3 months are defined as a blank period, at which time the onset of AA is not considered as a recurrence. This study did not believe that continued use of antiarrhythmic drugs was a relapse. Stroke (ischemic or hemorrhagic) can be diagnosed by cranial CT or MRI.

Secondary endpoints included death from various causes, major bleeding events, thromboembolism, repeated ablation, rehospitalization, discontinuation of oral anticoagulants, pericardial effusion, atrial esophageal fistula, and LAAO‐related events (occluder displacement, residual flow > 3 mm, and device‐related thrombosis).

### 2.5 Statistical analysis

Continuous variables were described as mean ± standard deviation (SD) if they conformed to a normal distribution, while those without a normal distribution were presented as median with interquartile ranges. Categorical variables were described as percentages (%). For survival analysis, the Kaplan–Meier estimate with a *p* value obtained with the log‐rank test analyzed the freedom from AA and major adverse cardiovascular events (MACE). Two‐sided *p* < .05 was considered significant for all analyses. The SAS 9.4 software (SAS Institute Inc.) was adopted to conduct all analyses.

## 3 RESULT

This study enrolled 138 patients undergoing the CA combined with LAAO procedure, including the DSA cohort (*n* = 71, 36 female, mean age of 71.8 ± 7.8) and the TEE cohort (*n* = 67, 26 female, mean age of 70.3 ± 8.7). The population had a high risk of stroke and bleeding, both of which the median CHA2DS2‐VASc score was 3.0 [2.0, 4.0] points and HAS‐BLED score was 2.0 [1.0, 3.0] points. Compared with the DSA cohort, the TEE cohort had a higher proportion of persAF [37 (55.2%) vs. 26 (36.6%)] and hemorrhage history [9 (13.4%) vs. 0]. No statistically significant differences in gender, age, body mass index, proBNP, eGFR, left atrial diameter, left ventricular ejection fraction, incidence of hypertension, diabetes, stroke, and medications were found. Detailed baseline characteristics are listed in Table 1.

**Table 1:** Baseline characteristics.

Parameters	Overall (*N* = 138)	DSA (*N* = 71)	TEE (*N* = 67)	*p* Value
Age, years	71.0 ± 8.3	71.8 ± 7.8	70.3 ± 8.7	0.244
Gender (female), *n* (%)	71 (51.5)	36 (50.7)	26 (38.8)	0.160
BMI	25.4 ± 3.9	25.6 ± 3.9	25.2 ± 4.0	0.559
AF type (persAF), *n* (%)	63 (45.7)	26 (36.6)	37 (55.2)	**0.028**
HAS‐BLED score	2.0 (1.0, 3.0)	2.0 (1.0, 3.0)	2.0 (1.0, 3.0)	0.557
CHA_2_DS_2_‐VASc score	3.0 (2.0, 4.0)	3.0 (2.0, 4.0)	3.0 (2.0, 4.0)	0.586
proBNP, pg/mL	749.0 (266.7, 1442.0)	749.0 (294.5, 1352.0)	784.1 (210.7, 1793.0)	0.960
eGFR, mL/(min × 1.73 m^2^)	86.0 ± 24.8	88.9 ± 24.6	82.9 ± 24.8	0.164
Left atrial diameter, mm	42.2 ± 4.7	42.2 ± 4.1	42.2 ± 5.2	0.966
LVEF, %	60.0 (56.0, 60.0)	60.0 (56.0, 60.0)	60.0 (55.0, 60.0)	0.502
Medical history				
Hypertension, *n* (%)	95 (68.8)	49 (69.0)	46 (68.7)	0.964
Diabetes mellitus, *n* (%)	33 (23.9)	20 (28.2)	13 (19.4)	0.228
Heart failure, *n* (%)	45 (32.6)	21 (29.6)	24 (35.8)	0.434
CHD, *n* (%)	26 (18.8)	13 (18.3)	13 (19.4)	0.870
Stroke history, *n* (%)	34 (24.6)	18 (25.4)	16 (23.9)	0.841
Hemorrhage history, *n* (%)	9 (6.5)	0	9 (13.4)	**0.004**
Medications				
OACs, *n* (%)	29 (21.0)	18 (25.4)	11 (16.4)	0.198
Warfarin, *n* (%)	7 (5.1)	4 (5.6)	3 (4.5)	1.000
NOACs, *n* (%)	22 (15.9)	14 (19.7)	8 (10.5)	0.130
Antiplatelet agents, *n* (%)	30 (21.7)	12 (16.9)	18 (26.9)	0.156

*Note*: Continuous variables are presented as mean ± SD, while categorical variables as percentage (%). Bold values are statistically significant.

Abbreviations: AF, atrial fibrillation; BMI, body mass index; CHD, coronary heart disease; eGFR, estimated glomerular filtration rate (calculated via CKD‐EPI formula); LVEF, left ventricular ejection fraction; NOAC, new oral anticoagulant; OAC, oral anticoagulant; persAF, persistent atrial fibrillation; proBNP, pro brain natriuretic peptide.

The procedure time of the DSA cohort was significantly reduced (95.7 ± 27.6 vs. 108.9 ± 30.3 min, *p* = .018), with a nonsignificant longer fluoroscopic time (15.2 ± 5.4 vs. 14.4 ± 7.1 min, *p* = .074). All of the combined procedures were performed successfully in both cohorts. The incidence of periprocedural complications including vagal reflex, phrenic nerve palsy, pericardial effusion, and cardiac tamponade did not reach statistical significance between the DSA cohort and the TEE cohort. There into, two patients were originally planned to undergo TEE evaluation during the procedure, but they could not tolerate it and switched to DSA for evaluation. Table 2 presents the periprocedural details of CA combined with LAAO.

**Table 2:** Periprocedural safety and efficacy evaluation between groups.

Procedural parameters	Overall (*N* = 138)	DSA (*N* = 73)	TEE (*N* = 65)	*p* Value
Procedural duration, min	101.2 ± 39.1	95.7 ± 27.6	108.9 ± 30.3	**0.018**
Fluoroscopic time, min	14.8 ± 7.8	15.2 ± 5.4	14.4 ± 7.1	0.074
LAA morphology				0.711
Chicken wing	36 (26.5)	17 (23.6)	19 (29.7)	
Cactus	40 (29.4)	24 (33.3)	16 (25.0)	
Windsock	30 (22.1)	15 (20.8)	15 (23.4)	
Cauliflower	30 (22.1)	16 (22.2)	14 (21.9)	
Landing zone, mm	21.1 ± 3.8	20.9 ± 4.2	21.1 ± 3.9	0.716
Occluder size (plug), mm	27.0 (24.0, 30.0)	27.0 (24.0, 30.0)	27.0 (24.0, 30.0)	0.574
Occluder plug size (pacifier), mm	24.0 (18.0, 24.0)	24.0 (18.0, 24.0)	24.0 (24.0, 30.0)	0.235
Occluder plate size (pacifier), mm	30.0 (28.0, 36.0)	30.0 (28.0, 30.0)	30.0 (28.0, 36.0)	0.439
Successful closure, *n* (%)	138 (100)	73 (100)	65 (100)	–
Occluder recapture, *n* (%)	7 (5.0)	2 (2.7)	5 (7.7)	0.350
Occluder resize, *n* (%)	4 (2.9)	0	4 (6.2)	0.101
Safety evaluation				
Vagal reflex, *n* (%)	2 (1.5)	2 (2.7)	0	0.528
Phrenic nerve palsy, *n* (%)	1 (0.7)	0	1 (1.5)	0.954
Residual flow, *n* (%)				
<3 mm	2 (1.5)	0	2 (3.1)	0.426
≥3 mm	0	0	0	–
Pericardial effusion, *n* (%)	2 (1.5)	0	2 (3.1)	0.426
Cardiac tamponade, *n* (%)	1 (0.7)	1 (1.4)	0	1.000

*Note*: Bold values are statistically significant.

Abbreviations: LAAO, left atrial appendage occlusion; PVI, pulmonary vein isolation; TSP, transseptal puncture.

**Figure 2:**
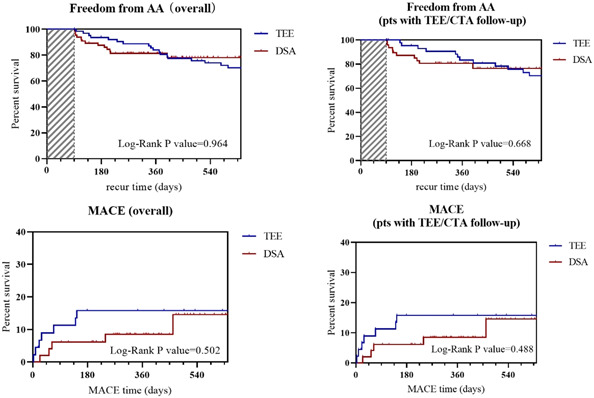
Freedom from AA and MACE by survival analysis. AA, atrial arrhythmia; DSA, digital subtraction angiography; MACE major adverse cardiovascular events; TEE, transesophageal echocardiography.

After an average of 24 months of clinical follow‐up, 10 patients were lost. Similar numbers of patients in the two cohorts underwent AA recurrence (13 [20.0%] in the DSA group and 18 [28.6%], *p* = .305) and stroke (*p* = .116). The incidence of death, whether all‐cause (1 [1.5%] in the DSA cohort and 2 [3.2%] in the TEE cohort, *p* = .616]) or due to cardiovascular disease (1 [1.6%] in the TEE cohort, *p* = .492), were similar. Further, there were similar numbers of patients in the two cohorts undergoing rehospitalization due to cardiovascular disease, with 12 (18.5%) in the DSA cohort and 19 (30.2%) in the TEE cohort, respectively (*p* = .15). The incidence of adverse events, including acute heart failure, pharmacy council of India (PCI), pacemaker implantation, major hemorrhage, and cardiac tamponade did not reach statistical significance between the DSA cohort and TEE cohort. Follow‐up TEE/CTA was performed in 96 patients (75.0%) detected similar composition of residual flow between the DSA and TEE cohort (3 cases of ≤3 mm residual flow in the TEE cohort, *p* = .62). Detailed information is described in Table 3. Supporting Information: Table 1 showed the safety and efficacy evaluation in patients completed CTA/TEE follow‐up. Kaplan–Meier estimates showed nonsignificant differences between the cohorts for freedom from AA (log‐rank *p* = .964) and MACE (log‐rank *p* = .502). The results of the survival analysis are shown in Figure 2.

**Table 3:** Follow‐up safety and efficacy evaluation.

Events	Overall (*N* = 128)	DSA (*N* = 65)	TEE (*N* = 63)	*p* Value
Efficacy evaluation				
AA recurrence, *n* (%)	31/128 (24.2)	13/65 (20.0)	18/63 (28.6)	0.305
Redo‐ablation, *n* (%)	4/128 (3.1)	2/65 (3.1)	2/63 (3.2)	1.000
Stroke/TIA, *n* (%)	3/128 (2.3)	0	3/63 (4.8)	0.232
Systemic thrombosis, *n* (%)	0	0	0	–
Safety evaluation				
All‐cause death, *n* (%)	3/128 (2.3)	1/65 (1.5)	2/63 (3.2)	0.616
Death due to cardiovascular disease, *n* (%)	1/128 (0.8)	0	1/63 (1.6)	0.492
Rehospitalization due to cardiovascular disease, *n* (%)	31/128 (24.2)	12/65 (18.5)	19/63 (30.2)	0.150
Acute heart failure, *n* (%)	13/128 (10.2)	6/65 (9.2)	7/63 (11.1)	0.777
PCI, *n* (%)	5/128 (3.9)	1/65 (1.5)	4/63 (6.4)	0.204
Pacemaker implantation, *n* (%)	7/128 (5.5)	5/65 (7.7)	2/63 (3.2)	0.440
Major hemorrhage, *n* (%)	2/128 (1.6)	1/65 (1.5)	1/63 (1.6)	1.000
Cardiac tamponade, *n* (%)	0	0	0	–
TEE/CTA follow‐up				
Completed	96 (75.0%)	50/65 (76.9)	46/63 (73.0)	**<0.001**
TEE, *n* (%)	73/96 (76.0)	31/50 (62.0)	42/46 (91.3)	
CTA, *n* (%)	23/96 (24.0)	19/50 (82.6)	4/46 (8.7)	
Displacement, *n* (%)	0	0	0	–
Residual flow, *n* (%)				
≤3 mm	3/96 (3.1)	0	3/46 (6.5)	0.620
>3 mm	0	0	0	–
Device related thrombosis, *n* (%)	0	0	0	–
Pericardial effusion, *n* (%)	0	0	0	–

*Note*: Bold values are statistically significant.

Abbreviations:  PCI, percutaneous coronary intervention; TEE, transesophageal echocardiography; TIA, transient ischemic attack.

## 4 DISCUSSION

In the present study, we first investigated the feasibility and safety of intraprocedural guidance of CA combined with LAAO procedure with DSA alone to TEE and DSA. By comparing their periprocedural and follow‐up clinical results, we concluded that intraprocedural DSA alone is not inferior to TEE with DSA evaluation for the feasibility and safety of the combined procedure.

TEE has been recommended by EHRA/EAPCI expert consensus as the standard method to guide LAAO procedures under general anesthesia.^6^ Although serious complications including esophageal perforation, esophageal injury, hematoma, laryngeal palsy, dysphagia, dental injury, or death, occur in less than 3% of TEE, the application of TEE is still limited in oral, esophageal, or gastric diseases.^21^ Moreover, general anesthesia not only increases the complexity and cost of the procedure but also has the potential transient of persistent cognitive dysfunction in the elderly.^22^

According to the various limitation of intraprocedural TEE guidance with general anesthesia, many centers have reported the feasibility and safety of applying DSA alone under local anesthesia without sedation to guide the LAAO procedure.^16^, ^17^, ^18^, ^19^ They mostly used the view of RAO/CAU and RAO/CRAN to measure LAA orifice, landing zone, and length to the greatest extent to avoid the PDL. In 2018, Yuniadi et al.^18^ compared the 36‐month follow‐up results of the LAAO procedure with the guidance of TEE and DSA in a small sample size, which proved the feasibility and safety of LAAO with DSA guided alone. Furthermore, Xiaochun Zhang et al.^19^ systematically analyzed the periprocedural and follow‐up clinical outcome of the evaluation of TEE and DSA in the LAAO procedure, and finally, they concluded that it is feasible to apply DSA alone to guide LAAO. As for safety, although DSA alone had no statistically significant difference in the incidence of intraprocedural complications and postprocedural follow‐up adverse events in LAAO patients compared with TEE with DSA, it still needed to be verified by more centers.

Although there have been no articles on the safety and feasibility of DSA alone to guide the combined procedure, the procedure time, fluoroscopy time, total radiation dose, and percentage of fluoroscopic PDL in the LAAO of our study is similar to the previous reports of the LAAO under procedural DSA guidance only.^16^, ^19^, ^23^ In the present study, compared with the TEE cohort, the procedure time of the DSA cohort was significantly reduced and there was no significant difference in the complication rate and fluoroscopy time. In the TEE cohort, two patients who could not tolerate TEE during the procedure were temporarily changed to the DSA cohort. This again illustrates the limitation of TEE in the patient's tolerance.

In our study, there were two patients with intraprocedural residual flow < 3 mm, and both patients were from the TEE cohort. Although the results are not statistically different, it is difficult to detect minor PDL under the guidance of the DSA and the detection rate may be lower than the actual incidence. Whether such the size of the PDL is meaningful has not been determined. Although the results of the clinical trial of LAAO indicate that a leak size of no more than 5 mm is considered safe, some reports of strokes related to smaller leaks have been described.^24^, ^25^ Regarding the classification of PDL, the mainstream view is that PDL size is categorized as minor (<3 mm), small (≥3–5 mm), moderate (≥5–9 mm), or large (≥10 mm). PDL caused by incomplete LAAO may promote blood flow stagnation, thrombosis, and embolism. The latest systematic review showed that LAAC patients with minor PDL could discontinue OAC and there had been no indications or data to support PDL closure, which required more research to verify.^26^

Compared with TEE, the main disadvantage of DSA is that it may underestimate device‐related thrombosis and minor PDL. Therefore, it is critical for them to take a standardized anticoagulation regimen after the procedure. In fact, on the basis of anticoagulation, device‐related thrombosis was a rare event.^27^ In this study, there have been no patients with device‐related thrombus under the detection of TEE/CTA in the DSA cohort. For minor PDL, there has been no conclusion on its harm.^26^ In addition to the guidance of the combined procedure, TEE can also be applied to evaluate acute left atrial ridge lesions after PVI. However, whether it is necessary to apply to evaluate such a situation remains to be discussed.^28^ The latest meta‐analysis in 2020 showed that intracardiac echocardiography (ICE) is as effective and safe as TEE in LAAO, and it also eliminates the need for general anesthesia and can be performed with local anesthesia. However, ICE requires separate intravenous access, which may increase the risk of vascular complications, and the high cost and additional equipment make many centers more inclined to other methods of intraprocedural guidance.^29^

## 5 LIMITATION

This was a single‐center nonrandom observational study with a limited number of patients, which may be biased. The significant difference in the proportion of persAF and hemorrhage history between the two cohorts partly affected the rigor of this study, although there was no significant difference in the important baseline characteristics that might affect the outcome between the two cohorts. In addition, the procedurers actually divided patients into either group according to their clinical experience with the preprocedural evaluation of TEE, which ultimately resulted in long TEE group procedure time and a low occluder recapture rate in the DSA group. Although a CHA2DS2‐VASc score was 3 and a HAS‐BLED score was 2, only 21% patients were on OAC in that we routinely recommended LAAO for patients who needed oral anticoagulation but have related contraindications or refused to take anticoagulant drugs, which may cause such bias. We did not find pericardial effusion, tamponade, and other complications due to our small sample size and the fact that some patients did not take TEE/CTA, so we cannot demonstrate the association between complications and the procedure. Importantly, during follow‐up, a significant higher proportion of patients in DSA group chose CTA for follow‐up examination rather than TEE. Previous study had validated that CTA could outperform TEE in examining LAAC device related events including PDL.^30^ The distinct difference in examination modality might cause underestimated device‐related events in TEE group. However, as the overall incidence of the events are relatively low, we failed to observe a significant difference in incidence of device‐related events. Studies with well designed follow‐up surveillance protocol are needed. What is more, the combined procedure in our study was performed mostly utilizing the cryoballoon ablation combined with LAA WATCHMAN™ device occlusion. Therefore, the promotion of DSA alone guidance required more research to verify.

## 6 CONCLUSION

Compared with DSA and TEE guidance, DSA‐guided combined procedure could shorten the procedural time while achieving similar periprocedural and long‐term feasibility and safety.

## CONFLICTS OF INTEREST STATEMENT

The author declare no conflict of interest.

## Supporting information

Supporting information.

## Data Availability

All data included in this study are available upon request by contact with the corresponding author.
