# Supplementary material for: Guiding atrial fibrillation ablation combined with left atrial appendage occlusion procedure by fluoroscopy with or without transesophageal echocardiography achieved comparable outcomes
Source: Clin Cardiol. 2023 Mar 9;46(5):549–57. doi: 10.1002/clc.23993 (PMC10189077; doi:10.1002/clc.23993)
Supplement: Supplementary file 1 — Supporting information. [file CLC-46-549-s001.docx]

**Supplementary table 1 Follow-up safety and efficacy evaluation in patients completed CTA/TEE.**

| Events | Overall  (N=96) | DSA  (N=50) | TEE  (N=46) | *P* value |
| --- | --- | --- | --- | --- |
| Efficacy evaluation | |  |  |  |
| AA recurrence, n (%) | 25/96 (26.0) | 11/50 (22.0) | 14/46 (30.4) | 0.347 |
| Redo-ablation, n (%) | 2/96 (2.1) | 1/50 (2.0) | 1/46 (2.2) | 1.000 |
| Stroke/TIA, n (%) | 3/96 (3.1) | 0 | 3/46 (6.5) | 0.620 |
| Systemic thrombosis, n (%) | 0 | 0 | 0 | - |
| Safety evaluation | |  |  |  |
| All-cause death, n (%) | 2/96 (2.1) | 1/50 (2.0) | 1/46 (2.2) | 1.000 |
| Death due to cardiovascular disease, n (%) | 1/96 (1.0) | 0 | 1/46 (2.2) | 1.000 |
| Rehospitalization due to cardiovascular disease, n (%) | 26/96 (27.1) | 11/50 (22.0) | 15/46 (32.6) | 0.243 |
| Acute heart failure, n (%) | 11/96 (11.5) | 5/50 (10.0) | 6/46 (13.0) | 0.883 |
| PCI, n (%) | 4/96 (6.5) | 1/50 (2.0) | 3/46 (6.5) | 0.551 |
| Pacemaker implantation, n (%) | 5/96 (5.2) | 3/50 (6.0) | 2/46 (4.4) | 1.000 |
| Major hemorrhage, n (%) | 2/96 (2.1) | 1/50 (2.0) | 1/46 (2.2) | 1.000 |
| Cardiac tamponade, n (%) | 0 | 0 | 0 | - |
| TEE/CTA follow-up |  |  |  |  |
| Displacement, n (%) | 0 | 0 | 0 | - |
| Residual flow, n (%) | |  |  |  |
| ≤3mm | 3/96 (3.1) | 0 | 3/46 (6.5) | 0.620 |
| >3mm | 0 | 0 | 0 | - |
| Device related thrombosis, n (%) | 0 | 0 | 0 | - |
| Pericardial effusion, n (%) | 0 | 0 | 0 | - |

TIA denotes transient ischemic attack, PCI percutaneous coronary intervention, TEE transesophageal echocardiography.
